# Tradeoffs in microbial carbon allocation may mediate soil carbon storage in future climates

**DOI:** 10.3389/fmicb.2013.00261

**Published:** 2013-09-04

**Authors:** Stephanie N. Kivlin, Bonnie G. Waring, Colin Averill, Christine V. Hawkes

**Affiliations:** Section of Integrative Biology, University of Texas at AustinAustin, TX, USA

**Keywords:** carbon allocation, extracellular enzyme activity, climate change, microbial physiology, biomass stoichiometry

Climate-induced changes in soil microbial physiology impact ecosystem carbon (C) storage and alter the rate of CO_2_ flux from soils to the atmosphere (Allison et al., 2010). The direction and magnitude of these microbial feedbacks depend on changes in saprotrophic bacterial and fungal C allocation in response to altered temperature, precipitation, and nutrient availability. Soil microbes may differentially allocate C in changing environments by altering processes such as enzyme production, C use efficiency (CUE), or biomass stoichiometry (Figure 1). However, because these mechanisms may operate simultaneously and interact, microbial physiological feedbacks on soil C storage are difficult to predict. For example, initial increases in microbial CUE or biomass C:N may be counteracted by increases in enzyme production to acquire limiting organic nutrients.

**Figure 1:**
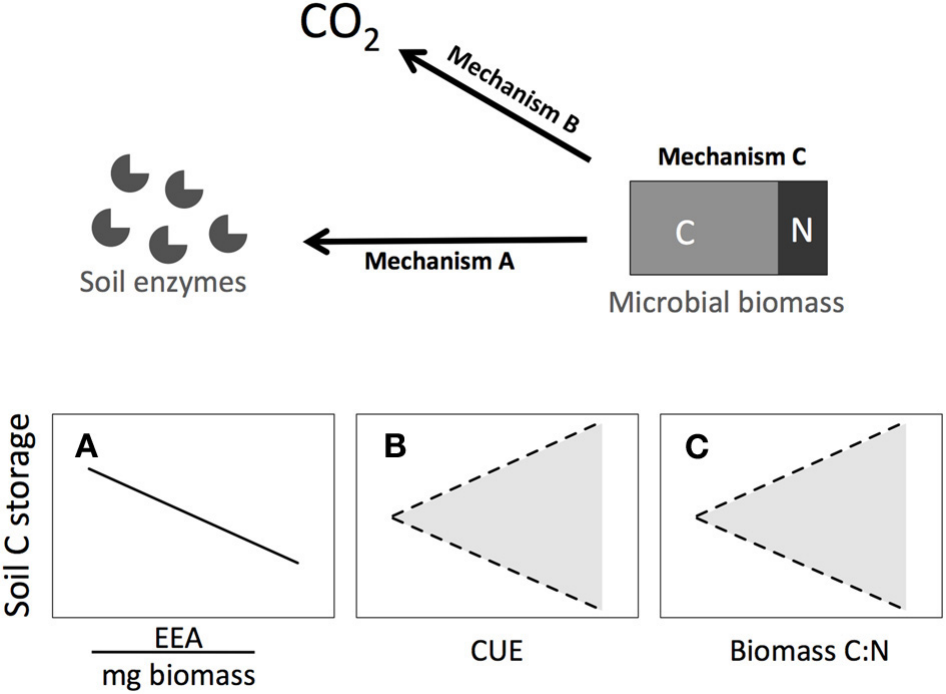
**Three mechanisms through which microorganisms can shift C allocation: (A)** extracellular enzyme activities, **(B)** carbon use efficiency, or **(C)** biomass stoichiometry. Each of these pathways can alter C storage in soils. Trend lines indicate expected responses when each mechanism is operating in isolation. In **(A)**, increased C allocation to extracellular enzymes can decrease soil C storage by enhancing C cycling rates. Shading in **(B** and **C)** shows uncertainty owing to expected interactions between the total microbial biomass pool, CUE, biomass C:N and enzyme production. Increased CUE or biomass C:N can augment soil C storage by increasing the amount of C retained in the soil microbial biomass. Alternatively, increased C in microbial biomass may cause microbes to produce more enzymes per unit biomass to acquire limiting organic nutrients, ultimately decreasing soil C stocks.

Few studies have standardized microbial process rates, such as extracellular enzyme production or respiration, to the size of the microbial biomass. Examining process rates alone may obscure the microbial physiological mechanisms that underlie climate-induced changes in soil C cycling, leading to contradictory patterns among different studies. For instance, in a large-scale survey of soil protease activities from climate manipulations, drier and warmer conditions resulted in lower extracellular enzyme activities (EEA) compared to ambient conditions (Brzostek et al., 2012). In contrast, drier soils have also been found to stabilize extracellular enzymes in water films, reducing enzyme turnover rates and increasing potential activities (Lawrence et al., 2009; German et al., 2012).

## Challenging paradigms

In this issue, Steinweg et al. (2013) examine the microbial mechanisms underlying ecosystem responses to climate change by quantifying soil EEA and microbial biomass under factorially manipulated precipitation (ambient, 50% of ambient, 150% ambient) and temperature [ambient, + 0.7 (low), + 2.05 (medium) and + 2.70°C (high)] treatments in the Boston-Area Climate Experiment. Overall, Steinweg and colleagues observed a trend for lower EEA per gram dry soil in drought and warming treatments relative to ambient conditions that was dependent on soil depth. However, mass-specific EEA was unaffected by climate manipulations except in June 2009, where elevated temperature led to higher EEA of all enzymes per unit microbial biomass in low and medium warming, but lower EEA per unit biomass at the highest warming level. Similarly, mass specific phosphatase and cellobiohydrolase EEAs were higher in drought treatments in June 2009.

These findings challenge our existing knowledge about the mechanisms driving EEA in soils. In the laboratory, EEA increases linearly with temperature over the narrow temperature range observed here (German et al., 2012). Steinweg and colleagues illustrate how considering EEA per unit microbial biomass can unveil a more complex relationship, with mass-specific EEA increasing with temperature up to a threshold around 2.2°C; well within the range of predicted warming over the next 100 years (IPCC, 2007). The effects of soil warming are often confounded by decreases in soil moisture in natural systems. Therefore, the non-linear correlations between EEA and soil temperature observed here and elsewhere (Allison and Treseder, 2008; Brzostek et al., 2012) may be driven by limitations on enzyme and substrate diffusion.

## Linking changes in microbial physiology with ecosystem biogeochemical cycles

The work of Steinweg and colleagues is an example of how shifts in gross process rates emerge from multiple interacting microbial mechanisms. However, these physiological feedbacks are often not incorporated into biogeochemical models. For instance, despite ample evidence that soil microbes shift allocation to enzyme production depending upon resource availability, EEA is often modeled simply as a function of the microbial biomass pool size. This approach may be valid in some ecosystems under steady state conditions or during the growing season (Kivlin and Treseder, 2013). However, complex interactions among changes in the proportion of active microorganisms under different temperatures and soil moistures can alter microbial biomass growth and enzyme allocation (Lennon and Jones, 2011), violating this assumption in altered climates. Based on the results of the current study, for example, traditional models may overestimate soil C storage under warming if they do not capture observed increases in mass-specific EEA, which could accelerate rates of soil C cycling over time. However, the shifts in mass-specific EEA appeared to be only seasonal and potentially could be counteracted by changes in total microbial biomass or CUE over the longer term. Microbial CUE, or ratio of growth to C uptake, is reduced under warming in both empirical tests and theoretical models (Manzoni et al., 2012; Tucker et al., 2013), yet this response can be mediated by changes in substrate recalcitrance (Frey et al., 2013) or microbial community composition (Bradford et al., 2008; Waring et al., 2013). Shifts in microbial CUE under altered precipitation regimes are more variable. Drought conditions can lead to higher microbial CUE over the short term, as osmolytes are accumulated in the microbial biomass (Manzoni et al., 2012), but repeated moisture pulses can cause decreases in CUE (Tiemann and Billings, 2011).

Shifts in intracellular C allocation may also affect element cycling at the ecosystem scale if the stoichiometric requirements of the microbial biomass are altered. Steinweg and colleagues found that enzyme ratios varied with season, indicating higher microbial C vs. N demand in the winter. They suggest that changes in enzyme stoichiometry may reflect increased microbial maintenance cost during freeze-thaw cycles, which impose a high C cost on the microbial biomass. Shifts in biomass C:N have also been observed in response to altered substrate stoichiometry (Fanin et al., 2013) and changes in community composition under drought (Cregger et al., 2012). Increasing biomass C:N may enhance soil C storage if biomass turnover is slow (Treseder et al., 2010) or if microbes synthesize extremely recalcitrant compounds that are difficult to decompose (Rillig et al., 2001). However, if microbial residues are decomposed more rapidly than plant litter inputs (e.g., Throckmorton et al., 2012) or larger microbial biomass C pools correspond to higher respiration rates over the long term, increases in the size or C content of the microbial biomass may actually enhance soil C loss.

## Future directions

Based on the findings of Steinweg et al. (2013) many of the parameters in current microbial physiology models may be better represented as allocation tradeoffs rather than constant values. In this instance, microbial allocation to resource acquisition (EEA) vs. growth may be dependent on the degree of environmental stress and biomass maintenance costs. Representing these parameters as functions rather than fixed values may enhance the predictive power of current soil C models, and increase their applicability to ecosystems where fewer parameters are known. Indeed, plant physiological models that incorporate C and resource allocation tradeoffs often perform better than either fixed-value or trait-based models [reviewed in Franklin et al. (2012)].

While Steinweg et al. (2013) focus on saprotrophic fungi and bacteria, physiological responses of mycorrhizal fungi to changing climates can also impact soil C storage (Clemmensen et al., 2013). Ecto- and ericoid mycorrhizal fungi can produce hydrolytic and oxidative enzymes, while arbuscular mycorrhizal fungi may produce hydrolytic phosphatases (Tisserant et al., 2012). All mycorrhizal fungi likely invest more in enzymes to acquire nutrients rather than C, as C is provided by the host plant (Smith and Read, 2008; Rineau et al., 2012). Similar to saprotrophs, mycorrhizal fungi can shift C allocation between biomass and respiration in response to altered environmental conditions, and appear to have decreased CUE under novel temperature and moisture, but not fertilization (Johnson et al., 2002; Heinemeyer et al., 2006, 2007; Hawkes et al., 2008). However, more research into the tradeoffs between growth, respiration and EEA for mycorrhizal fungi is needed, as mycorrhizal fungi have been identified as the dominant pathway by which recently fixed C enters soils in several systems (Godbold et al., 2006; Clemmensen et al., 2013).

Multiple processes will have large consequences for soil C storage in future climates, including climate controls on enzyme production and turnover, and tradeoffs in microbial allocation to growth, respiration, and resource acquisition. Yet, because all of these processes interact, the effects of climate change on soil C pools and fluxes can be extremely variable. As Steinweg and colleagues demonstrate, measuring mass-specific microbial responses is the first step toward improving our understanding of microbial physiological responses to altered climate regimes. However, studies that simultaneously examine the links among these mechanisms will be necessary to predict when tradeoffs in microbial C allocation occur and their long-term effects on soil C storage. By viewing ecosystem responses to temperature and precipitation through the lens of microbial physiology, we may arrive at a more mechanistic understanding of soil feedbacks on climate change.
